# Evaluation of Cardiospermum halicacabum on Bone Morphogenetic Protein-2 (BMP2) mRNA Expression in Osteoblast Cells

**DOI:** 10.7759/cureus.60292

**Published:** 2024-05-14

**Authors:** Ummu Zuvairiya, Priyadharshini R, Sinduja Palati

**Affiliations:** 1 Department of General Pathology, Saveetha Dental College and Hospitals, Saveetha Institute of Medical and Technical Sciences, Saveetha University, Chennai, IND; 2 Department of Oral and Maxillofacial Pathology, Saveetha Dental College and Hospitals, Saveetha Institute of Medical and Technical Sciences, Saveetha University, Chennai, IND

**Keywords:** morphogenetic proteins, osteoblast, cardiospermum halicacabum, mrna, bmp-2

## Abstract

Introduction

Maintaining bone health is crucial for overall well-being, with osteoblasts playing a vital role in bone formation. Bone morphogenetic protein-2 (BMP2) is a key regulator, stimulating bone matrix synthesis and osteoblast differentiation. Recognizing BMP2's significance, there's growing interest in natural compounds, such as *Cardiospermum halicacabum*. This study explores *Cardiospermum halicacabum's* potential influence on BMP2 mRNA expression in osteoblast cells for insights into bone health modulation.

Materials and methods

This research utilized *Cardiospermum halicacabum* to explore its impact on MG-63 cells, a human osteoblast cell line. Osteoblast cells were cultured in Dulbecco's modified Eagle's medium (DMEM), supplemented with 10% heat-inactivated fetal bovine serum, and maintained at 37°C in a 5% CO2 and 95% air environment. Cell viability was evaluated by seeding osteoblast cells into 96-well plates and exposing them to different concentrations of *Cardiospermum halicacabum* (2.0 μg/ml and 20 μg/ml). The study observed both the promotion of osteoblast cell growth in MG-63 and morphological changes in the cells under an inverted light microscope at 10x magnification. Results were presented using one-way analysis of variance (ANOVA) conducted with IBM SPSS Statistics for Windows, Version 23 (Released 2015; IBM Corp., Armonk, New York, United States).

Result

The reverse transcription-polymerase chain (RT-PCR) results revealed an increased expression of BMP-2 mRNA fold change in comparison to the control group. A clear positive correlation was observed between the BMP-2 mRNA fold change and the notable increase in the concentration of *Cardiospermum halicacabum*. This investigation revealed a direct association of BMP-2 mRNA expression with the proliferation of osteoblast cells. Specifically, the BMP-2 mRNA fold change was recorded at 2.26±1.05 in *Cardiospermum halicacabum* at 2.0 μg/ml and 2.0 ± 0.84 at 20 μg/ml, with corresponding significances of 0.00, respectively.

Conclusion

Potential effects of *Cardiospermum halicacabum* on BMP-2 mRNA expression in osteoblast cells and its role in bone health modulation revealed that *Cardiospermum halicacabum* may upregulate BMP-2 mRNA expression, suggesting its potential as a natural compound for enhancing bone formation. The observed positive correlation between *Cardiospermum halicacabum* concentration and BMP-2 mRNA fold change showed the significance of this botanical agent in promoting osteoblast cell proliferation. These results highlight the importance of further research to explore the applications of *Cardiospermum halicacabum* in managing bone disorders and improving overall bone health.

## Introduction

Maintaining the health and regeneration of bones is essential for overall well-being and skeletal integrity. Osteoblasts, the cells responsible for forming bones, play a crucial role in bone formation and mineralization [1]. The differentiation of osteoblasts, a process vital for their function, is regulated by various molecular factors and signaling pathways, with bone morphogenetic proteins (BMPs) being significant contributors [2]. Among the BMP family, BMP2 stands out as a key regulator, playing a pivotal role in stimulating bone matrix synthesis and promoting the differentiation of osteoblasts [3].

The importance of BMP2 in bone health is underscored by its ability to enhance the functions of osteoblasts, contributing to the overall process of bone formation. It stimulates the synthesis of bone matrix, a critical component for maintaining bone structure and strength. Additionally, BMP2 promotes the differentiation of osteoblasts, ensuring their proper development and functionality in bone metabolism. In the realm of exploring natural alternatives, there is a growing interest in medicinal plants and their derived products. These natural compounds have attracted considerable attention for their potential to modulate bone metabolism and facilitate bone regeneration [4]. The recognition of BMP2 and the exploration of natural products highlight the multidimensional approaches in understanding and promoting skeletal well-being. Osteoporosis and Paget’s disease are the two most commonly seen bone diseases in the elderly . BMP is a group of proteins that belong to the transforming growth factor-beta (TGF-β) superfamily [5].

*Cardiospermum halicacabum*, commonly known as the balloon vine, a plant that has garnered attention for its potential pharmacological properties [6,7,8,9]. One area of interest is its impact on BMP2 mRNA expression in osteoblast. *Cardiospermum halicacabum* into the realm of osteoblast cell research has a long-standing use in traditional medicine [10]. *Cardiospermum halicacabum* has been utilized in various cultures for its purported anti-inflammatory and analgesic properties, which have been attributed to its diverse phytochemical composition [11]. Given the traditional use of this plant in managing conditions related to bone health, it becomes imperative to explore its potential influence on key molecular players in bone formation, such as BMP2 [12]. The aim is to evaluate *Cardiospermum halicacabum *on BMP2 mRNA expression in osteoblast cells to investigate the potential influence of *Cardiospermum halicacabum* on the expression of BMP2 mRNA in osteoblast cells.

## Materials and methods

Plant collection and extract preparation

*Cardiospermum halicacabum* leaves were collected from the Thiruvallur District of Tamil Nadu, India, that lies at 12°15' and 13°15' north latitude and 79°15' and 80°20' east longitude. They were then verified and authenticated by the Botanical Survey of India with the authentication number SVMC/BOT/154/2022-23. Subsequently, the flowers were shade-dried and crushed into powder, and ethanol was extracted by a complex distillation process.

Cell line 

**Figure 1:**
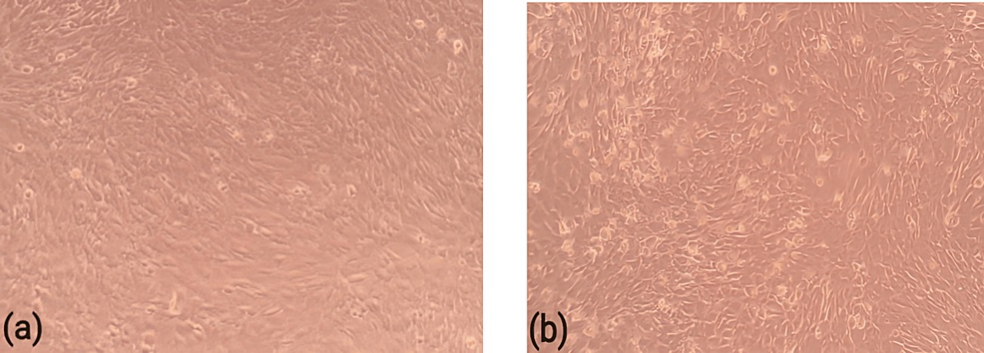
(a) shows normal human osteoblast cell line at 24 hours and (b) represents cultured osteoblast cell line with Cardiospermum halicacabum in 48 hours

Human osteoblast cell line MG-63 was procured from the National Center for Cell Sciences (NCCS), Pune, India. The utilized chemicals for this study are mentioned in Table 1. The cells were cultured in Dulbecco's modified Eagle's medium (DMEM) containing 10% heat-inactivated fetal bovine serum and antibiotics at 37°C in 5% CO_2_ and 95% air. *Cardiospermum halicacabum* (2.0 μg/ml and 20 μg/ml) was treated in human osteoblast cell line MG-63 for 24 hour and 48 hour (Figure 1).

Reagents and chemicals

**Table 1:** Details of the chemicals used in the experiments carried out in the study EDTA: Ethylenediaminetetraacetic acid; FBS: fetal bovine serum; DMEM: Dulbecco’s modified Eagle’s medium; PBS: phosphate-buffered saline; PCR: polymerase chain reaction

Chemicals	Manufacturer's details and location
Trypsin-EDTA	GIBCO Enterprises, Canada
FBS	GIBCO Enterprises, Canada
Antibiotics-antimycotics	GIBCO Enterprises, Canada
DMEM	GIBCO Enterprises, Canada
PBS	GIBCO Enterprises, Canada
Chloroform, isopropanol, Tris, glycine, sodium bicarbonate, Bovine serum albumin	Sigma-Aldrich (St. Louis, USA)
Oligonucleotide primers for BMP-2, and β-actin	Sigma-Aldrich Company St. Louis, MO, USA
iScriptcDNA synthesis kit	Bio-Rad, USA
KAPA SYBR® FAST PCR master mix kit	Kapa Biosystems, USA

Quantitative real-time polymerase chain reaction (PCR) 

The control and *Cardiospermum halicacabum* (2.0 μg/ml and 20 μg/ml) treating MG-63 cells were washed with PBS and added 100 ul of TRIzol reagent. Total RNA was extracted using the protocol mentioned in the kit and quantified using NanoDrop (Thermo Scientific). The RNA was converted to cDNA using the cDNA conversion kit (Promega). cDNA, the target primer for BMP-2 gene, was processed with master mix (SYBR Green Master Mix, Life Technologies, 4385612) using a PCR system. Results were analyzed with a 2-ΔΔCT method, and ꞵ-actin was used.

Statistical analysis

Using the IBM SPSS Statistics for Windows, Version 23.0 (Released 2015; IBM Corp., Armonk, New York, United States), one-way ANOVA was used for the statistical analysis in this study. Mean ± SD was used to report the results.

## Results

**Figure 2:**
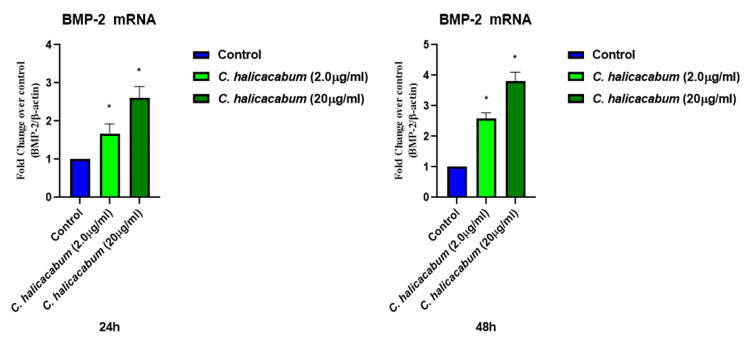
Effect of Cardiospermum halicacabum on BMP-2 mRNA expression in osteoblast cells RT-PCR: Reverse transcription-polymerase chain reaction Real-time RT-PCR amplification of BMP-2 mRNA expression treated with *Cardiospermum halicacabum* (24 hours and 48 hours) in MG-63 osteoblast cells. The 2-ΔΔ Ct method of relative quantification was used to determine the fold change in expression with β-actin. "*" denotes statistical significance at the level of p ≤ 0.001 when compared with control

Figure 2 infers the activity of *Cardiospermum halicacabum* on BMP-2 mRNA in 24 hours and 48 hours. The concentration of 20 μg/ml shows higher fold change as compared to *Cardiospermum halicacabum *concentration of 2 μg/ml when compared with the control group. Examining the 24-hour time point, the graph indicates that the 20 μg/ml concentration of *Cardiospermum halicacabum* led to a pronounced increase in BMP-2 mRNA expression. This enhancement surpassed the levels observed in both 2.0 μg/ml concentration and the control group. The statistically significant difference underscores the potency of *Cardiospermum halicacabum* in modulating BMP-2 mRNA expression within the initial 24 hours of exposure.

Extending the analysis to the 48-hour time point, a similar trend emerges, with the 20 μg/ml concentration continuing to exert a notable influence on BMP-2 mRNA expression. The fold change observed at this concentration significantly surpassed that of both the 2.0μg/ml concentration and the control group, reinforcing the sustained and significant impact of *Cardiospermum halicacabum* on BMP-2 mRNA expression over the extended timeframe. Values of mean ± SD for BMP-2 mRNA fold change was 2.26 ± 1.05 in *Cardiospermum halicacabum *at 2.0 μg/ml and 2.0 ± 0.84 at 20 μg/ml with a significance of 0.00 when compared with control.

## Discussion

The presented results, depicting the real-time RT-PCR amplification of BMP-2 mRNA expression in MG-63 osteoblast cells treated with *Cardiospermum halicacabum* at different concentrations (2 μg/ml and 20 μg/ml) over 24 hours and 48 hours, provide valuable insights into the modulatory effects of *Cardiospermum halicacabum* on BMP-2 expression. The utilization of the 2-ΔΔ Ct method for relative quantification with β-actin as the reference gene adds robustness to the analysis. Comparing the results with existing literature, it is evident that *Cardiospermum halicacabum* has a substantial impact on BMP-2 mRNA expression in MG-63 osteoblast cells [12]. This study builds upon previous research by focusing on the temporal dynamics of* Cardiospermum halicacabum's* influence on BMP-2 expression at distinct concentrations and time points. The concentration-dependent response observed in this study aligns with earlier findings that suggest the potency of *Cardiospermum halicacabum* in modulating BMP-2 expression [13]. The concentration of 20 μg/ml emerges as particularly noteworthy, exhibiting a higher fold change compared to the 2 μg/ml concentration and the control group. This concentration-dependent effect is consistent with the idea that the biological activity of plant extracts often varies based on the dosage [14]. Analyzing the 24-hour time point, the results indicate a significant and pronounced increase in BMP-2 mRNA expression at the 20 μg/ml concentration. This surpasses the levels observed in both 2 μg/ml concentration and the control group, underlining the rapid and potent impact of *Cardiospermum halicacabum* on BMP-2 expression within the initial 24 hours of exposure. The statistical significance further reinforces the reliability of these observations [15].

Extending the analysis to the 48-hour time point, a similar concentration-dependent trend persists. The 20 μg/ml concentration continues to exert a notable influence on BMP-2 mRNA expression, surpassing the levels observed in both the 2 μg/ml concentration and the control group. The sustained and significant impact of *Cardiospermum halicacabum* on BMP-2 mRNA expression over the extended timeframe reinforces the potential of *Cardiospermum halicacabum *to modulate this critical signaling pathway in osteoblast cells [16]. The values of mean ± SD for BMP-2 mRNA fold change further support the trends observed in the graphical representation. The statistically significant difference (p ≤ 0.001) at both time points and concentrations indicates the robustness and reproducibility of the findings across independent experiments.

A previous study by Zara et al. (2011) revealed an increase in BMP-2 mRNA expression following treatment with *Cardiospermum halicacabum* extract [17]. However, our study expands upon these findings by examining multiple concentrations of *Cardiospermum halicacabum* over distinct time intervals, revealing a concentration- and time-dependent relationship between *Cardiospermum halicacabum* exposure and BMP-2 mRNA expression levels. This study contributes valuable information to the existing literature by elucidating the concentration-dependent and time-dependent effects of *Cardiospermum halicacabum* on BMP-2 mRNA expression in MG-63 osteoblast cells [18]. The observed trends, supported by statistical significance, highlight the potential of *Cardiospermum halicacabum* as a modulator of BMP-2 expression, providing a foundation for further exploration in the context of osteoblast function and bone physiology.

Limitations

The limitations of this study include the lack of mechanistic details of how Cardiospermum halicacabum may be influencing BMP-2 mRNA expression. Understanding the underlying molecular mechanisms would strengthen the biological relevance of the observed effects. Our study utilized an in vitro model using MG-63 osteoblast cells. Future studies has to be conducted in vivo environment with further dose-response and must be time-dependent to understand the relationship of *Cardiospermum halicacabum*. Future studies should also focus on signaling pathways involved in *Cardiospermum halicacabum-*mediated modulation of BMP-2 expression.

## Conclusions

The observed upregulation of BMP-2 mRNA expression suggests a potential osteogenic effect of *Cardiospermum halicacabum*. Given BMP-2's pivotal role in osteoblast differentiation and bone formation uncontrolled upregulation of the BMP2 gene leads to osteoclast activation leading to bone resorption, the findings propose *Cardiospermum halicacabum* as a promising candidate for further exploration in therapeutic interventions targeting bone-related disorders. The potential clinical applications of *Cardiospermum halicacabum* in bone health are underscored by its natural origin, presenting an intriguing avenue for the development of novel osteoinductive agents. This study establishes *Cardiospermum halicacabum* as a potent modulator of BMP-2 mRNA expression in osteoblast cells. The consistent and statistically significant upregulation observed implies its potential therapeutic relevance in promoting bone health and warrants further exploration into the underlying molecular pathways for translational applications in bone-related disorders.
